# Variety and Dynamics of Proteoforms in the Human Proteome: Aspects of Markers for Hepatocellular Carcinoma

**DOI:** 10.3390/proteomes5040033

**Published:** 2017-11-23

**Authors:** Stanislav Naryzhny, Victor Zgoda, Artur Kopylov, Elena Petrenko, Olga Kleist, Аlexander Archakov

**Affiliations:** 1 Institute of Biomedical Chemistry, Pogodinskaya 10, Moscow 119121, Russia; vic@ibmh.msk.su (V.Z.); a.t.kopylov@gmail.com (A.K.); el.petrenko@bk.ru (E.P.); archak@ibmc.msk.ru (A.A.); 2 Petersburg Nuclear Physics Institute, National Research Center “Kurchatov Institute”, Gatchina, Leningrad Region 188300, Russia; okleyst@mail.ru

**Keywords:** protein species/proteoform, dynamics, abundance, 2DE, proteome, ESI LC-MS/MS, biomarker

## Abstract

We have previously developed an approach, where two-dimensional gel electrophoresis (2DE) was used, followed by sectional analysis of the whole gel using high-resolution nano-liquid chromatography-mass spectrometry (ESI LC-MS/MS). In this study, we applied this approach on the panoramic analysis of proteins and their proteoforms from normal (liver) and cancer (HepG2) cells. This allowed us to detect, in a single proteome, about 20,000 proteoforms coded by more than 4000 genes. A set of 3D-graphs showing distribution of these proteoforms in 2DE maps (profiles) was generated. A comparative analysis of these profiles between normal and cancer cells showed high variability and dynamics of many proteins. Among these proteins, there are some well-known features like alpha-fetoprotein (FETA) or glypican-3 (GPC3) and potential hepatocellular carcinoma (HCC) markers. More detailed information about their proteoforms could be used for generation of panels of more specific biomarkers.

## 1. Introduction

Today, the main aim in human proteomics is a complete catalogue of all human proteins. As the human genome is deciphered this task becomes clear and purposeful; if there is a protein-coding gene a corresponding protein should be found. Until now, this survey of proteins was performed mainly using a bottom-up approach that includes protein digestion and subsequent mass spectrometric analysis of the peptides produced [1]. The situation in proteomics is much more complicated as proteins can exist as different forms (protein species or proteoforms) [2,3]. In an alternate approach, a detailed analysis of these proteoforms can be done using a top-down approach, where a whole protein is analysed [4,5]. Because of the variety of proteoforms and their range of concentrations (7–8 orders of magnitude) their identification and quantitation is a challenge that is difficult to attain. Recently, we described a combination of top-down and bottom-up approaches to optimize proteomic analysis [6,7,8]. To obtain information about different proteoforms, not just proteins, a sectional analysis of 2DE gels, or so called “pixel-based approach” in combination with ESI LC-MS/MS was used [6,7,8]. We applied this approach to study the human proteome using the cancer cell line HepG2 and normal human liver tissue. Such comparative analysis can be beneficial for obtaining a dynamic profile of the human proteome and revealing possible cancer biomarkers. As hepatocellular carcinoma (HCC) is one of the leading causes of cancer-related deaths worldwide, there is a need for novel biomarkers to increase the sensitivity and specificity of analysis for early HCC diagnosis [9,10,11]. More detailed analysis of proteomes can give us more information about the rearrangements of proteins that happens during cancerogenesis. This information can be used to identify new biomarkers [12,13]. Recently, an excellent in-depth comparative and quantitative proteomic analysis of human adult hepatocytes and HepG2 cells was performed by Wiśniewski et al. [14,15]. Our study, though not as detailed in the number of proteins analyzed, is complementary to this study by broadening the set of different proteoforms detected.

## 2. Materials and Methods 

Human cells (hepatocellular carcinoma, HepG2) were cultured under standard conditions [16] [17,18]. To prepare samples for protein extraction, the cells were detached with 0.25% Trypsin-EDTA solution, washed 3 times with PBS, and treated with Rabillound lysis buffer (7 M urea, 2 M thiourea, 4% CHAPS, 1% DTT, 2% ampholytes, pH 3–10, protease inhibitor mixture) [17,18]. Liver tissue samples were provided within the framework of collaboration with the Chromosome-Centric Human Proteome Project (C-HPP). Extraction was performed by lysis after grinding the tissue in liquid nitrogen according to two-dimensional electrophoresis (2DE) protocol described in [19]. All procedures for 2DE were carried out as described previously [7,20,21]. Gels were stained with Coomassie Blue R350, scanned by ImageScanner III (GE Healthcare, Pittsburgh, PA, USA) and analysed using ImageMaster 2D Platinum 7.0 (GE Healthcare) [22]. The tryptic peptides from crude lysates for MS analysis were obtained using a protocol for filter-aided sample preparation (FASP) [23]. Mass spectrometry was performed according to the protocol for ESI LC-MS/MS described elsewhere [6,22]. Proteolysis was performed by incubation with trypsin (“Trypsin Gold”, 10 µg/mL) at least 4 h at 37 °C. Tryptic peptides were dissolved in 5% (*v/v*) formic acid. MS/MS analysis was carried out in duplicate on an Orbitrap Q-Exactive Plus (Thermo Scientific, Waltham, MA, USA). Exponentially modified PAI (emPAI) defined as the number of identified peptides divided by the number of theoretically observable tryptic peptides for each protein was used to estimate protein abundance [24,25].

## 3. Results

**Figure 1:**
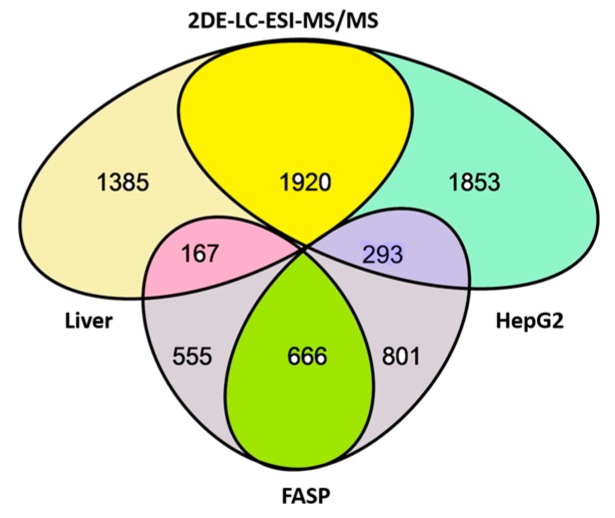
Overlap of proteins (genes) detected in liver and HepG2 extracts by FASP protocol (bottom ellipses) and by 2DE gel sectional analysis using ESI LC-MS/MS (top ellipses).

We have performed a panoramic study of human proteins and their proteoforms using a cancer cell line (HepG2) and normal liver tissue. Previously, some of these data were already published [7,19]. We generated the list of proteins identified in liver and HepG2 cell extracts using treatment with trypsin according to the FASP protocol [23], and by separation according to pI/Mw using 2DE, followed by sectional analysis of the gel by ESI LC-MS/MS. A total of 20,462 proteoforms encoded by 3773 genes were identified in the case of HepG2 cells [7], and 14,667 proteoforms, encoded by 3305 genes, in the case of liver cells [19]. Here, we present further analyses of these data. The basic information about the number of proteins detected by these methods is presented in Figure 1.

In the bottom, the number of proteins (genes) detected by shotgun mass-spectrometry (1221) using FASP protocol (left ellipse (1221): the liver, right ellipse (1467): HepG2 cells) is presented. Only 666 proteins were detected in both liver and HepG2 cells, while 555 proteins were detected only in liver, and 801were detected only in HepG2 cells. This is because of the level of detection sensitivity in our experiment and the levels of proteins in liver and HepG2 cells. The quantity of some proteins is enough to be detected in both samples, but some are only detected in liver and not in HepG2 cells and vice versa. This statement is confirmed by experiments using sectional analysis (top ellipses), when many more proteins were detected. Using sectional analysis, a total of 1920 proteins were detected in both liver and HepG2 cells (including many that were only detected in liver or HepG2 cells before). Again, many proteins were detected in liver only (1385) or HepG2 cells (1853). Concerning sensitivity, it is relevant to stress that only 293 proteins were detected in HepG2 cells but not in liver (in reverse case, 167) using both types of experiments. That confirms our statement about the sensitivity issue. Additionally, it is interesting to compare our data with data published in the paper by Wiśniewski et al. [14]. It happens that most of the abovementioned 293 proteins (detected in HepG2 cells only) were also identified by Wiśniewski et al. [14]. Furthermore, they showed that their level is much higher in HepG2 cells than in hepatocytes. Interestingly, despite the greater sensitivity of detection and the larger number of proteins detected by Wiśniewski et al. [14], they did not detect 30 of these 293 proteins (Supplementary Table S1).

The main part of this study is a set of proteoform profiles that we generated based on a combination of 2DE with LC ESI-MS/MS. We have produced these profiles as 3D graphical images. Some profiles are very similar in both samples and contain only one or two proteoforms (peaks). Often, proteins have many proteoforms, and the profiles for some of them are very different in liver and HepG2 cells. The most abundant peak usually has pI/Mw coordinates that are congruent with theoretical ones. The profiles of some proteins have an exceptionally large amount of proteoforms. Mostly, these are samples from HepG2 cells. Keeping in mind the cancerous nature of these cells, we have paid special attention to proteins that already are used or are under consideration to be used as tumor biomarkers. It is of note that the list of such markers is actually very long [26]. Since our object here is HCC, we narrowed the analysis on biomarkers for this tumor (Table 1).

**Figure 2:**
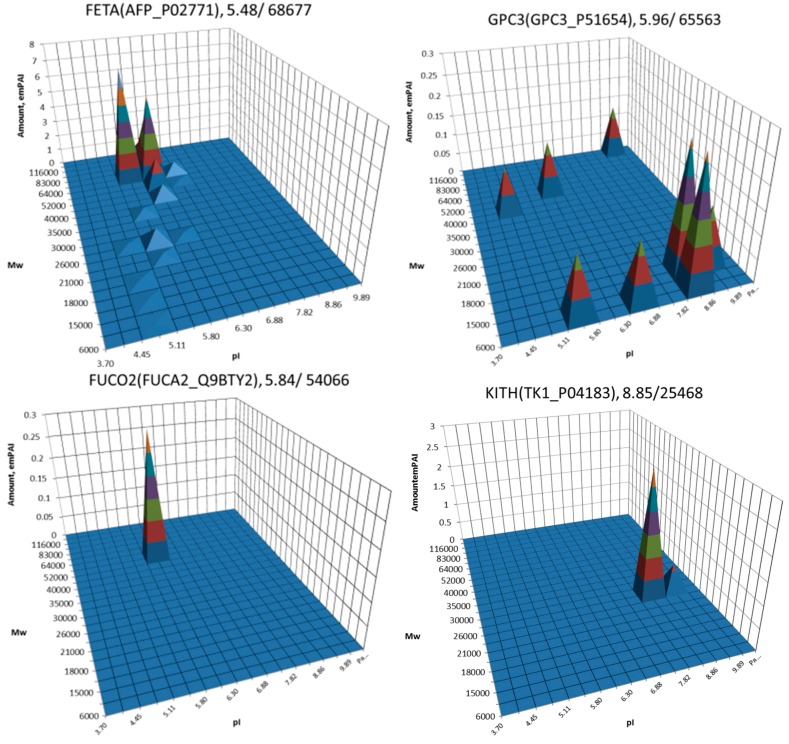

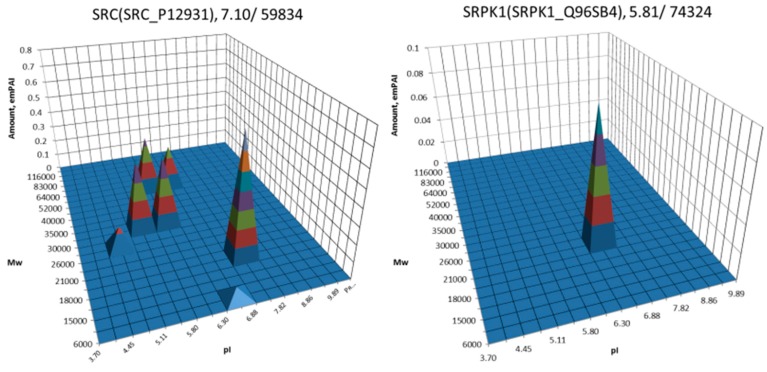
Profiles of some biomarkers from Table 1 detected in HepG2 cells but not in liver. On the top of each graph we show the protein name, Uniprot number and theoretical pI/Mw. The coordinates are: isoelectric point (pI), molecular weight (Mw), protein abundance (emPAI).

**Figure 3:**
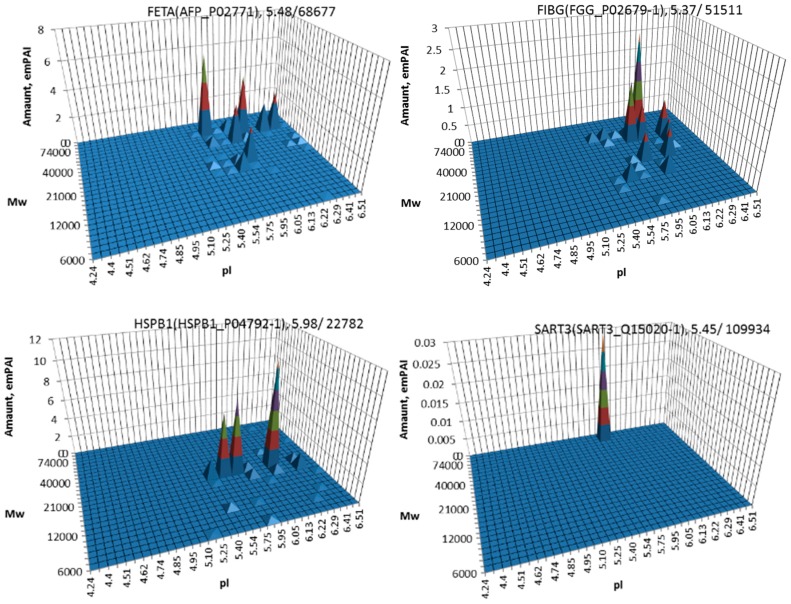
Profiles of alpha-fetoprotein (FETA), fibrinogen (FIBG), heat shock protein beta 1 (HSPB1), and squamous cell carcinoma antigen (SART3) in HepG2 cells obtained using higher resolution sectional analysis (pI 4-7, gel 24 × 20 cm, 252 sections).

**Figure 4:**
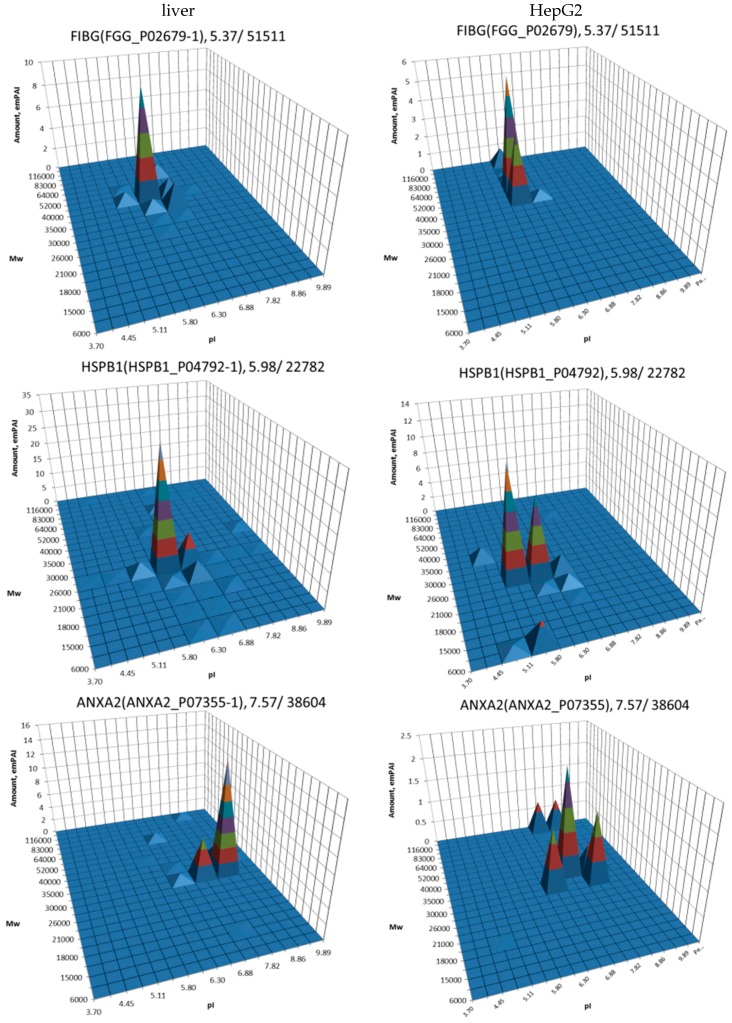

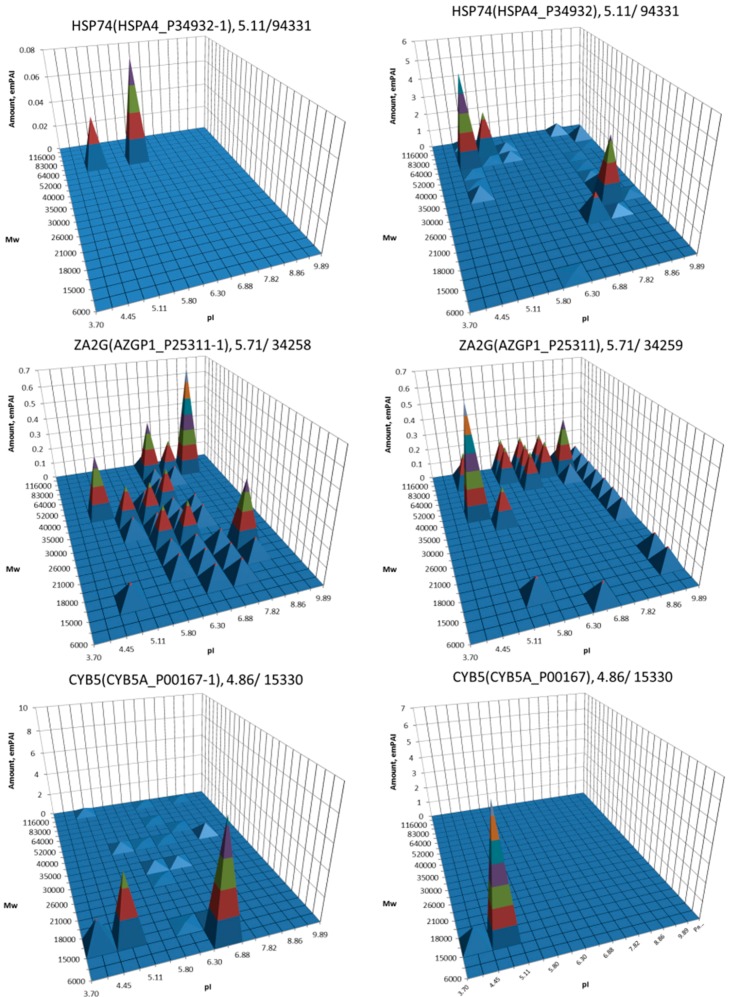

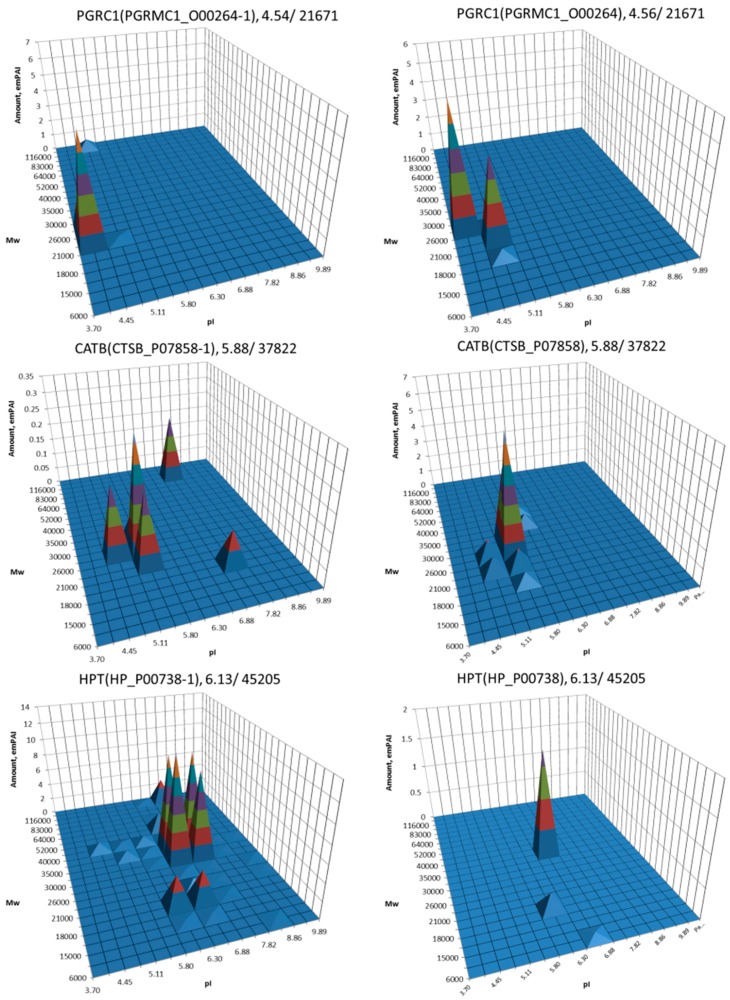
Profiles of biomarkers from Table 1 detected in HepG2 cells and in liver. Left: liver, right: HepG2 cells. On the top of each graph we show the protein name, Uniprot number, and theoretical pI/Mw. The coordinates are: isoelectric point (pI), molecular weight (Mw), protein abundance (emPAI).

The most well-known protein and the only one approved for clinical usage as a marker for HCC is alpha-fetoprotein (FETA) [27,28]. FETA levels in serum may increase with hepatocyte regeneration and in case of development of HCC [29]. It remains the most commonly used screening biomarker for HCC [10,28]. However, increased serum levels of FETA might be a result of other liver deceases (hepatitis, liver cirrhosis etc.) decreasing the specificity of FETA testing for HCC. Furthermore, FETA is not expressed at high levels in all HCC patients, resulting in decreased sensitivity. Importantly, while FETA protein is not always a good marker for HCC, there is an example of a more specific proteoform which is used as a biomarker. A fucosylated form of serum AFP is most closely associated with HCC. This proteoform is designated as AFP-L3 and used as a more specific biomarker for HCC [30]. In our case, 18 proteoforms of FETA were detected in HepG2 extracts (Figure 2). Even more proteoforms (35) were observed when sectional analysis with higher resolution was applied (Figure 3). In liver extract, this protein was not detected with a great enough reliability (at least two significant sequences). That confirms its usage as a HCC biomarker. There are more proteins from the list of HCC biomarkers (Table 1) that were detected in HepG2 cells only (GPC3, FUCO2, KITH, SRC, SRPK1) (Figure 2). Other proteins were detected in both samples (Figure 4). For instance, profiles of heat shock protein beta (HSPB1) or fibrinogen gamma chain (FIBG) are very similar, but HSP74, ANXA2, ZA2G, CYB5, PGRC1, CATB, HPT are different. In all cases, we can find many proteoforms presented in HepG2 cells but not in liver and vice versa. For instance, in the case of haptoglobin (HPT), which exhibits decreased levels in HCC [11], we observed a strong simplification of the profile in HepG2 cells compared to liver (Figure 4). In the case of heat shock protein beta 1 (HSPB1) and annexin A2 (ANXA2), profiles are very similar in liver and HepG2 cells, but with a clear anodic shift of peaks in HepG2 cells which may be due to phosphorylation, as phosphorylation is a known PTM for these proteins [41,42]). Zinc-alpha-2-glycoprotein (ZA2G) is characterized by a set of different proteoforms (more than 30) distributed all around the gel. Many of these proteoforms have a greater Mw compared to the theoretical Mw (this protein can be heavily glycosylated [43]). 

## 4. Discussion

In general, profiles of proteins in HepG2 cells tend to have more proteoforms (peaks) than in liver, but opposite cases are also observed. According to the positions of the peaks, preliminary assumptions about their origins can be made. For instance, the lower weight proteoforms can be products of proteolysis, which itself can be of different nature (post-translational proteolytic processing, degradation, regulation etc.). The proteoforms having similar Mw but more acidic pI, compared to the theoretical pI, can be the products of PTMs that add negative or remove positive charges (phosphorylation, acetylation, methylation etc.). The shift to the basic direction can be a result of carboxyl group modifications (amidation, esterification). Some PTMs leading to a big shift in pI, as well as in Mw, are ubiquitination, SUMOylation, or glycosylation. Splice variation is another reason to produce peaks located differently from the canonical sequence pI/Mw position. Such situations should not have a great impact as we have been showing only proteoforms of the same isoforms in the graphs. Taken together, we cannot specify the type of modifications but only give an estimation (by pI/Mw) of these proteoforms. For more specific evaluation of proteoforms a detailed analysis of each profile should be performed.

There is a resolution challenge in our experiments. On average, we found that every protein profile in liver and in HepG2 cells has five proteoforms. It is necessary to remind the reader that a single PTM, like acetylation, methylation or phosphorylation can produce a pI shift close to 0.05 [44,45]. However, in our experiments, the size of gel sections in the pI range is much bigger (0.7–0.8). This means that we are missing many cases of slight modifications and detect only heavy modifications. Actually, this is a limited technical restriction and the resolution can be improved by running bigger gels and analyzing smaller gel sections (Figure 3), but it will dramatically increase the time and effort required.

## 5. Conclusions

A combination of top-down proteomics (2DE separation of proteins) with bottom-up proteomics allows very convenient visual representations of information about diverse proteoforms coded by the same genes (proteoform profiles). Here, we have compared more than 1100 profiles of the most abundant proteins in liver and HepG2 cells. Among them, we analyzed profiles of known and potential HCC markers that could be helpful to further improve the specificity of testing for this disease. In case of alpha-fetoprotein (FETA), we found its presence in HCC as multiple proteoforms. There is a chance that among these there could be forms more specific than AFP-L3. Profiles of heat shock protein beta 1 (HSPB1) and annexin 2 (ANXA2) in HepG2 compare to liver cells are characterized by pronounced shifts of proteoforms towards more acidic pIs. It usually happens in case of phosphorylation or acetylation, and these proteins are known for these PTMs. Accordingly, these proteoforms could be better HCC markers than the levels of HSPB1 and ANXA2.

## Figures and Tables

**Table 1:** A list of known and potential biomarkers for hepatocellular carcinoma.

No.	Gene	UniProt	Protein Name	Reference
1	AFP	P02771	Alpha-fetoprotein	[10,11,27,28,29,30,31,32,33]
2	GPC3	P51654	Glypican-3	[10,11,33]
3	F2	P00734	Prothrombin	[10,11]
4	SPP1	P10451	Osteopontin	[28,34]
5	HSPB1	P04792	Heat shock protein beta-1	[11,33]
6	HSPA4	P34932	Heat shock 70 kDa protein 4	[33]
7	FUCA2	Q9BTY2	Plasma alpha-l-fucosidase	[35]
8	SART3	Q15020	Squamous cell carcinoma antigen recognized by T-cells 3	[36]
9	GOLM1	Q8NBJ4	Golgi membrane protein 1	[37]
10	ANXA2	P07355	Annexin A2	[27]
11	AZGP1	P25311	Zinc-alpha-2-glycoprotein	[33]
12	SRC	P12931	Proto-oncogene tyrosine-protein kinase Src	[38]
13	SRPK1	Q96SB4	SRSF protein kinase 1	[39]
14	FGG	P02679	Fibrinogen gamma chain	[40]
15	PGRMC1	O00264	Membrane-associated progesterone receptor component 1	[11]
16	CYB5A	P00167	Cytochrome b5	[11]
17	CTSB	P07858	Cathepsin B	[11]
18	HP	P00738	Haptoglobin	[11]
19	TK1	P04183	Thymidine kinase, cytosolic	[35]

## Supplementary Materials

The following are available online at www.mdpi.com/2227-7382/5/4/33/s1. Table S1: Proteins detected in HepG2 only.

## Abbreviations

The following abbreviations are used in this manuscript:

2DE	two-dimensional electrophoresis
ESI LC-MS/MS	liquid chromatography-electrospray ionization-tandem mass spectrometry
HCD	higher energy collisional dissociation
ABC	ammonium bicarbonate
CAN	acetonitrile
PTM	post-translation modification
emPAI	exponential modified form of protein abundance index
HCC	hepatocellular carcinoma
